# Assessment of blood and urine total antioxidant and oxidative status in alcohol and acetone fatal poisonings

**DOI:** 10.1038/s41598-025-09494-x

**Published:** 2025-07-07

**Authors:** Iwona Ptaszyńska-Sarosiek, Miłosz Nesterowicz, Edyta Gołaś, Anna Niemcunowicz-Janica, Anna Zalewska, Małgorzata Żendzian-Piotrowska, Mateusz Maciejczyk

**Affiliations:** 1 Department of Forensic Medicine, Medical University of Bialystok, Waszyngtona 13, 15-269 Białystok, Poland; 2 Students Scientific Club “Biochemistry of Civilization Diseases” at the Department of Hygiene, Epidemiology and Ergonomics, Medical University of Bialystok, Mickiewicza 2C, 15-022 Białystok, Poland; 3 Department of Restorative Dentistry, Medical University of Bialystok, Skłodowskiej-Curie 24A, 15-276 Białystok, Poland; 4 Department of Hygiene, Epidemiology and Ergonomics, Medical University of Bialystok, Mickiewicza 2C, 15-022 Białystok, Poland

**Keywords:** Biomarkers, Pathogenesis

## Abstract

Heavy alcohol consumption is a common cause of fatal poisoning, unlike acetone poisoning, which is very rare. Although oxidative stress plays a crucial role in alcohol and acetone toxicity, no studies compare the total redox status in fatally poisoned individuals. The total antioxidant capacity (TAC), total oxidative status (TOS), and oxidative stress index (OSI) were examined in the blood and urine of patients fatally poisoned with ethanol (n = 21) and acetone (n = 21), along with healthy controls (n = 21). The TAC, TOS, and OSI concentrations were determined by means of colorimetric methods, and the statistical analysis was conducted through non-parametric tests. The blood TAC and TOS were significantly increased in the ethanol- and acetone-intoxicated cadavers as compared to the controls. The blood TAC levels above 55.12 nmol/mg of protein differentiate alcohol- and acetone-intoxicated subjects from the control group with a high sensitivity and specificity. The blood TAC was also positively correlated with the alcohol (r = 0.504), acetone (r = 0.476), and isopropanol blood levels (r = 0.514). However, the TAC, TOS, and OSI in the blood were poorly correlated with the corresponding urine concentrations. The urinary TAC in the ethanol-poisoned subjects was effectively enhanced in comparison to the control and acetone-poisoned subjects. The urinary TAC was also positively associated with the urine alcohol concentration (r = 0.413) and negatively correlated with the acetone (r = − 0.453) and isopropanol urine levels (r = − 0.36). Fatal alcohol and acetone intoxications disrupt the circulating redox status, favoring antioxidant reactions. Differences in the blood and urine redox homeostasis may be due to the organ toxicity of alcohol and acetone.

**Supplementary Information:**

The online version contains supplementary material available at 10.1038/s41598-025-09494-x.

## Introduction

Fatal ethanol poisoning, also known as acute alcohol poisoning, is a severe medical condition caused by excessive consumption of pure ethanol or alcoholic beverages^1^. In the United States, an average of 2,221 deaths from alcohol poisoning occurred per year between 2010 and 2012, translating to about 8.8 deaths per million population, mostly among men aged 35–64^2^. Ethanol, commonly found in beer, wine, and liquor, is a central nervous system (CNS) depressant that may impair brain function and lead to respiratory failure, coma, and even death^1^. Ethanol inhibits neurotransmission in the brain by increasing the activity of gamma-aminobutyric acid (GABA) receptors and decreasing the activity of glutamate receptors. It slows down the brain function and weakens the body’s response^3,4^. In the initial stage of intoxication, a person may experience euphoria, disorientation, as well as motor imbalance. As the concentration of alcohol in the blood increases, the symptoms worsen and may include nausea, vomiting, breathing problems, unconsciousness, and seizures^5^. The critical blood alcohol concentration, that may lead to death, varies depending on the body’s tolerance and other factors such as weight, age, gender, and also health. The blood ethanol concentration of 4‰ or more is considered potentially fatal^6^.

Acetone is a common chemical compound found in many everyday products, such as nail polish removers, paint thinner, and cleaners. It is also used in the chemical industry as a solvent and in the manufacturing of many plastics and other materials^7^. Despite its widespread use, acetone may become fatal to humans under certain circumstances. Acetone poisoning occurs when a person ingests or inhales high levels of that chemical substance, potentially causing life-threatening effects on the body’s organs and systems^7^. Fatal acetone poisoning is extremely rare^8^. According to the data from the National Poison Data System (NPDS), only one single case of fatal acetone (single-substance) intoxication was reported in the entire United States between 2018 and 2022^9^. Acetone is rapidly absorbed through the mucous membranes of the respiratory tract, gastrointestinal tract, and skin. Once in the bloodstream, acetone may depress the CNS, leading to symptoms such as a headache, dizziness, confusion, as well as an impaired motor coordination. In the cases of severe poisoning, convulsions, a loss of consciousness, and a respiratory failure may occur. Acetone is also metabolized in the liver to acetic acid, which may lead to metabolic acidosis and have severe consequences for the cardiovascular system and other organs. In addition, high concentrations of acetone may damage cell membranes, leading to tissue necrosis and damage to internal organs^7,10^. In the case of acetone, the blood concentration of at least 0.55‰ is considered lethal^6^.

Ethanol and acetone significantly impair the redox balance. Ethanol primarily interacts with the liver’s metabolic pathways, where it is metabolized to acetaldehyde by alcohol dehydrogenase (ADH) and subsequently to acetic acid by aldehyde dehydrogenase (ALDH)^11^. That metabolic process leads to the generation of reduced NADH. A decreased NAD^+^/NADH ratio may impair the mitochondrial function and disrupt the balance between oxidative and reductive reactions, contributing to metabolic disorders such as fatty liver disease and lactic acidosis^12^. On the other hand, acetone is a ketone body that undergoes metabolic conversion into acetoacetate and subsequently into β-hydroxybutyrate. Those metabolites play crucial roles in the cellular energy production by participating in the Krebs cycle, a central metabolic pathway that generates ATP^10^. Beyond its role in the energy metabolism, β-hydroxybutyrate also influences the cellular redox homeostasis by modulating the formation of reactive oxygen species (ROS). ROS overproduction may induce oxidative stress overwhelming antioxidant defense mechanisms^7,13,14^. However, the exact effects of alcohol and acetone poisoning on the cellular redox homeostasis remain to be unknown. Little is also known about the use of redox biomarkers to assess alcohol and acetone toxicity.

The significant health hazards linked to ethanol and acetone poisoning highlight the need to identify reliable biomarkers that may help estimate the likelihood of mortality. Proficient biomarkers could facilitate a prompt identification, more focused actions, and more efficient treatment plans for affected individuals. The search for biomarkers that reflect the biological changes associated with acute ethanol and acetone intoxication is still ongoing. Given the critical contribution of oxidative stress to ethanol and acetone toxicity, redox biomarkers may help assess xenobiotic exposure^7,12–14^. Both measurements of antioxidants and compounds formed through the reaction of free radicals with the body’s cellular components are used for determining the redox homeostasis. Due to the plethora of enzymatic and non-enzymatic antioxidants, as well as products resulting from the oxidation of proteins, lipids, and DNA, some alternatives may arise from the assessment of the total antioxidant capacity (TAC), total oxidative status (TOS), and the oxidative stress index (OSI). A measure of the redox homeostasis may be the sum of all antioxidants (TAC) or oxidants (TOS) in the biological material. The OSI is the ratio of TOS to TAC, indicating the direction in which the oxidation–reduction balance is shifting^15–20^.

This study examines the TAC, TOS, and OSI to assess ethanol and acetone toxicity.

## Materials and methods

### Cadavers

The Local Ethical Committee of the Medical University of Bialystok (Poland) approved the research (no. R-I-002/82/2013). The number of patients was determined based on similar studies and is typical for forensic medicine^21–24^.

Three groups, each consisting of 21 deceased people, were studied. Concentrations of ethanol, acetone, and isopropanol were determined in each cadaver (Table 1; Table S1). In the first one – the control group, abrupt causes of death included chest injuries (6 individuals, 28.57%) and brain injuries (15 persons, 71.43%). At the crime site, the fatalities happened immediately and without any delay. All of the people from the second group died as a result of ethanol intoxication (blood concentration of at least 4‰)^6^. Acetone poisoning was the cause of death in the third group (blood concentration of at least 0.55‰)^6^. Cases with concurrent hypothermia were excluded. Every cadaver in the second and third group had atrophy of the cerebrum and focal or diffuse steatosis of the liver. The bodies were taken straight to the morgue once they had been pronounced dead. Pathological alterations in other organs were excluded in all of the groups. Diseases such as cancer and chronic inflammatory conditions as well as other illnesses or harmful behavior, like smoking, were also excluded. Family members attested to the absence of drug misuse. The corpses were preserved at the temperature of 4 °C in the refrigerator before the postmortem analysis. Biological material was collected during forensic autopsies carried out at the Department of Forensic Medicine of the Medical University of Bialystok (Poland). The autopsy was performed each time by the same experienced pathologist (I.P.-S.).

**Table 1:** Blood and urine concentrations of ethanol, acetone, and isopropanol in control, alcohol- and acetone-intoxicated cadavers: median, minimal, and maximal values.

	Control group (*n* = 21)			Alcohol poisoning (*n* = 21)			Acetone poisoning (*n* = 21)		
	Median	Minimum	Maximum	Median	Minimum	Maximum	Median	Minimum	Maximum
Sex (male/female)	12/9			12/9			12/9		
Age [years]	51	27	67	51	27	67	51	27	67
Blood alcohol concentration [‰]	0	0	0	4.1	4	5	0.4	0	3
Blood acetone concentration [‰]	0	0	0	0	0	0	0.6	0.55	2.35
Blood isopropanol concentration [‰]	0	0	0	0	0	0	0.13	0	0.6
Urinary alcohol concentration [‰]	0	0	0	4.4	4	6.2	1	0	3.1
Urinary acetone concentration [‰]	0	0	0	0	0	0	0.73	0.55	1.57
Urinary isopropanol concentration [‰]	0	0	0	0	0	0	0.17	0	0.9

Twelve hours following the death, sampling was conducted during a medico-legal autopsy. 5 mL samples of blood from the femoral vein as well as urine from the bladder were drawn using a syringe. The biological samples underwent centrifugation at 3000 × g for 20 min at 4 °C. Before the biological analysis, the supernatant had been separated, frozen, and kept in Eppendorf tubes at − 80 °C. By means of headspace gas chromatography (HS-GC) with flame ionization detection (FID), the levels of ethanol, acetone, as well as isopropanol were ascertained. The chromatograph (Thermo Electron Corporation Trace GC Ultra; Waltham, MA, USA) with an automated headspace injector and an FID detector was used. Both ZB-BAC1 (film thickness: 30 m × 0.32 mm ID × 1.8 µm) and ZB-BAC2 (30 m × 0.32 mm ID × 1.2 µm) capillary columns were included. The carrier gas was helium at 1.8 mL/min. The following temperatures were set: 40 °C for the column, 150 °C for the injector, 200 °C for the detector, 60 °C for sample heating, and 5 min for the thermoregulation period. A range of 0.2 to 4‰ was found on the standard curve for all three analytes.

### Redox status

All reagents were purchased from Sigma-Aldrich (Saint Louis, MO, USA): 2,2′-azino-bis(3-ethylbenz-thiazoline-6-sulfonic acid) (ABTS; as diammonium salt; cat. no. A1888), 6-hydroxy-2,5,7,8-tetramethylchroman-2-carboxylic acid (Trolox; cat. no. 238813), ferrous ammonium sulfate (as hexahydrate; cat. no. 203505), glacial acetic acid (cat. no. 33209-M), glycerol (cat. no. 15523-M), hydrogen peroxide (H_2_O_2_; as a solution; cat. no. H1009), o-cresosulfonphthalein-3,3-bis(sodium methyliminodiacetate) (xylenol orange; as tetrasodium salt; cat. no. 398187), o-dianisidine dihydrochloride (cat. no. D3252), sodium acetate (as a solution; cat. no. 71196), sodium chloride (NaCl) (cat. no. S9888), sulfuric acid (H_2_SO_4_; as a solution; cat. no. 30743-M). Type I reagent-grade deionized water was utilized. Using a multimode microplate reader (Tecan Infinite M200 PRO; Männedorf, Switzerland), absorbance measurements were made.

Erel’s colorimetric method was used for determining the total antioxidant capacity (TAC), in which the cationic radical ABTS (ABTS^·+^) was neutralized by antioxidants contained in the sample. The Trolox standard curve was used for calculating the TAC concentration that was reported in terms of µmol Trolox/mg of total protein. Each well received the following additions: 200 µL of R1 solution (0.4 M acetate buffer, pH 5.8), 5 µL of the test sample, and 20 µL of R2 solution (ABTS^·+^ in 30 mM acetate buffer, pH 3.6). Before having combined R1 and R2 to create the sample blank, the initial absorbance had been determined. The wavelength at which the absorbance measurements were made was 660 nm. Subsequently, the chemicals were mixed promptly, and the measurement was conducted again following a 5-min incubation^15,25,26^.

Erel’s colorimetric method was used for measuring the total oxidative status (TOS), in which oxidants in the sample converted Fe^2^⁺ to Fe^3^⁺ ions. Next, Fe^3^⁺ ion detection was performed using xylenol orange. An H_2_O_2_-based calibration curve was used for determining the TOS concentration that was reported in terms of nmol of H_2_O_2_ equivalent/mg of total protein. 225 µL of R1 solution (150 μM xylenol orange, 140 mM NaCl, and 1.35 M glycerol in 25 mM H_2_SO_4_, pH 1.75), 35 µL of the test sample, and 11 µL of R2 solution (5 mM Fe^2^⁺ and 10 mM o-dianisidine in 25 mM H_2_SO_4_) were added to each well. Prior to having combined R1 and R2, the original absorbance had been measured to serve as the sample blank. The absorbance measurements were made using a bichromatic approach, with the primary wavelength of 560 nm and the secondary one of 800 nm. The chemicals were mixed instantly, and the measurement was repeated after an incubation period of five minutes^19,26,27^.

The ratio of TOS to TAC was used for computing the oxidative stress index (OSI)^25^.

The bicicinchoninic acid (BCA) method was used for quantifying the total protein content using a Thermo Scientific PIERCE BCA Protein Assay commercial kit (Rockford, IL, USA).

### Statistical analysis

All the results were standardized to the total protein content. GraphPad Prism 10 (GraphPad Software, La Jolla, CA, USA) and Past 4.13 (Øyvind Hammer, Oslo, Norway) were used for the purpose of the statistical analysis. It was decided to use the Kruskal–Wallis ANOVA test as there was no normal distribution. Dunn’s post-hoc test was employed for a more thorough analysis and multiple-adjusted p-value was calculated. The results were displayed in terms of percentiles and median values (minimum–maximum). In order to analyze the relationship between variables, the non-parametric Spearman rank-order correlation coefficient was used. The diagnostic value of the TAC, TOS, and OSI was estimated by means of the receiver operating characteristic (ROC) analysis. For every parameter, the best cut-off values and the area under the curve (AUC) were found, guaranteeing good specificity and sensitivity. In every test, the statistical significance was *p* < 0.05.

## Results

### Blood

**Fig. 1:**
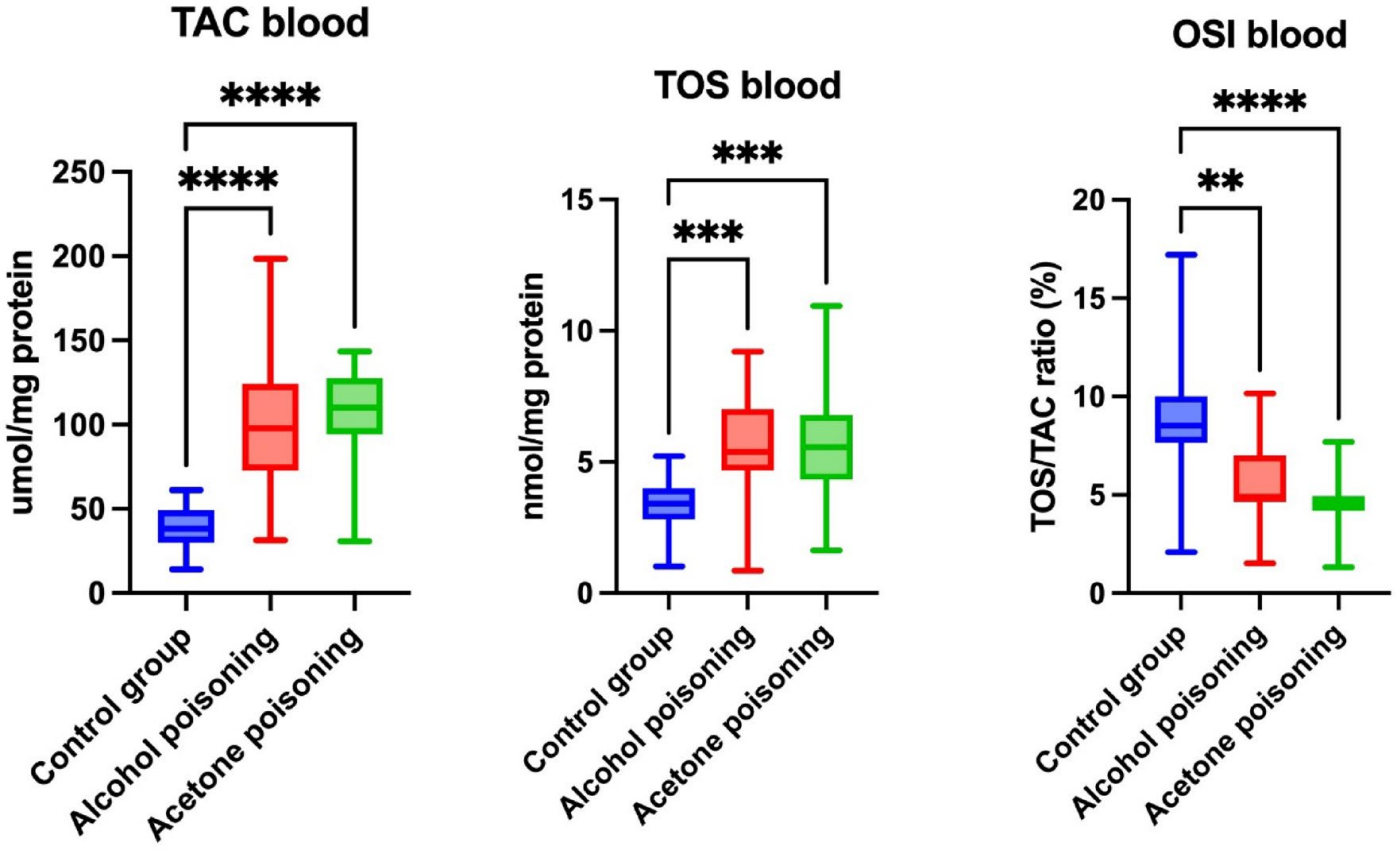
Blood levels of total antioxidant capacity (TAC), total oxidative status (TOS), as well as oxidative stress index (OSI) in control (blue bars), fatal ethanol poisoning (red bars), and fatal acetone poisoning (green bars) groups. OSI: oxidative stress index; TAC: total antioxidant capacity; TOS: total oxidant status; **p < 0.01; ***p < 0.001; ****p < 0.0001.

The blood TAC level was markedly increased in the ethanol (+ 214.14%, *p* < 0.0001) and acetone poisoning (+ 252.56%, *p* < 0.0001) groups when compared to the controls. The TOS level in the blood was also significantly higher in the both experimental groups (+ 136.53%, *p* = 0,0001 and also + 132.45%, *p* = 0,0003, respectively) versus the control group. However, the blood value of the OSI was markedly lower in the ethanol- (− 42.54%, *p* = 0,001) and acetone-poisoned cadavers (− 63.25%, *p* < 0.0001) than in the control individuals (Fig. 1).

### Urine

**Fig. 2:**
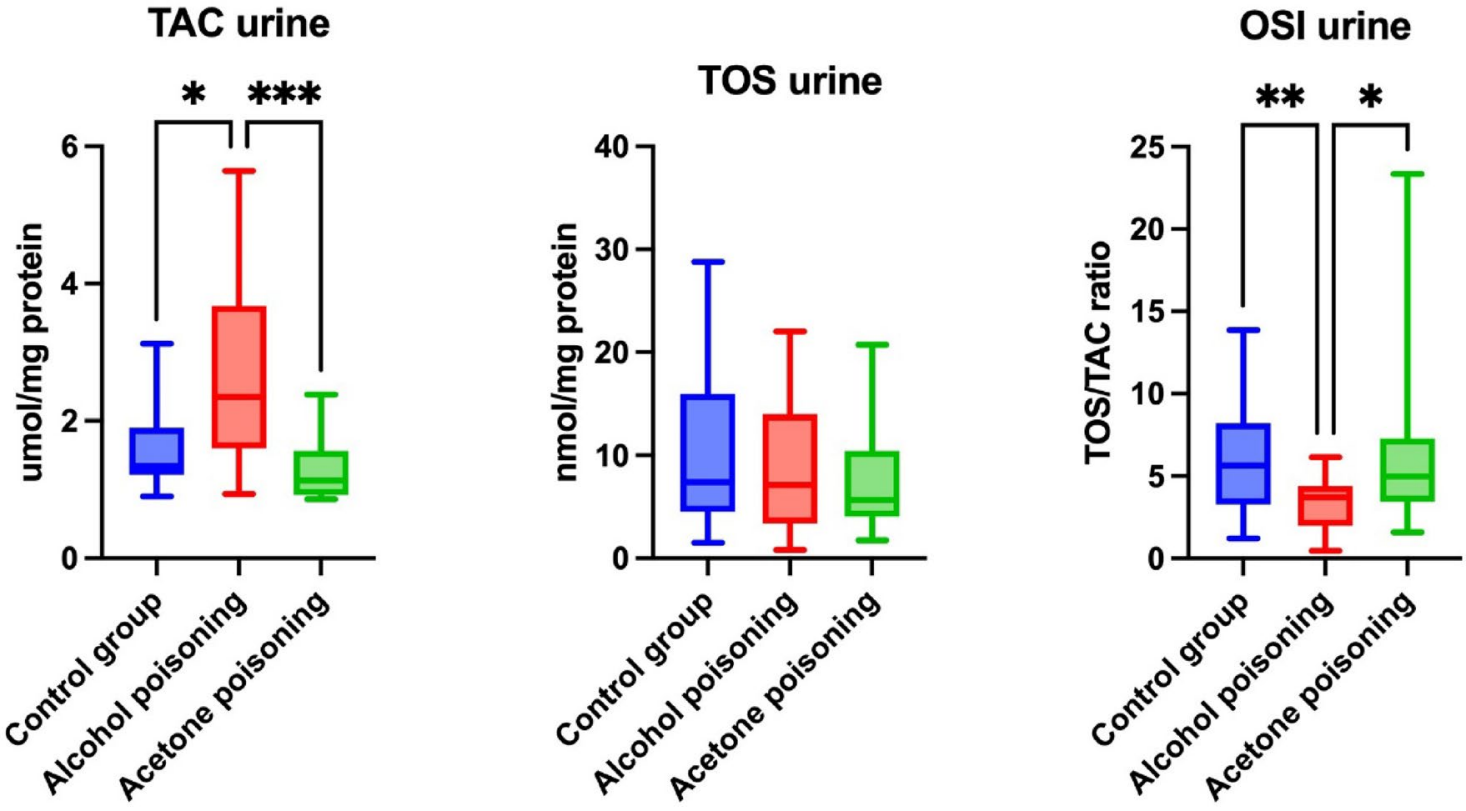
Urinary levels of total antioxidant capacity (TAC), total oxidative status (TOS), as well as oxidative stress index (OSI) in control (blue bars), fatal ethanol poisoning (red bars), and fatal acetone poisoning (green bars) groups. OSI: oxidative stress index; TAC: total antioxidant capacity; TOS: total oxidant status; *p < 0.05; **p < 0.01; ***p < 0.001.

The urinary level of the TAC was effectively enhanced in the ethanol poisoning versus the control group (+ 48.32%, *p* = 0.036). The parameter in the acetone-poisoned individuals was relevantly reduced in comparison with the ethanol-poisoned ones (− 53.8%, *p* = 0.0001). There were no significant differences between the groups as far as the urinary TOS levels were concerned. The urinary OSI value was markedly suppressed in the ethanol poisoning compared to the control group (− 46.49%, *p* = 0.007). The biomarker was substantially higher in the acetone- than in the ethanol-poisoned cadavers (+ 75.72%, *p* = 0.0268) (Fig. 2).

### Correlations

**Fig. 3:**
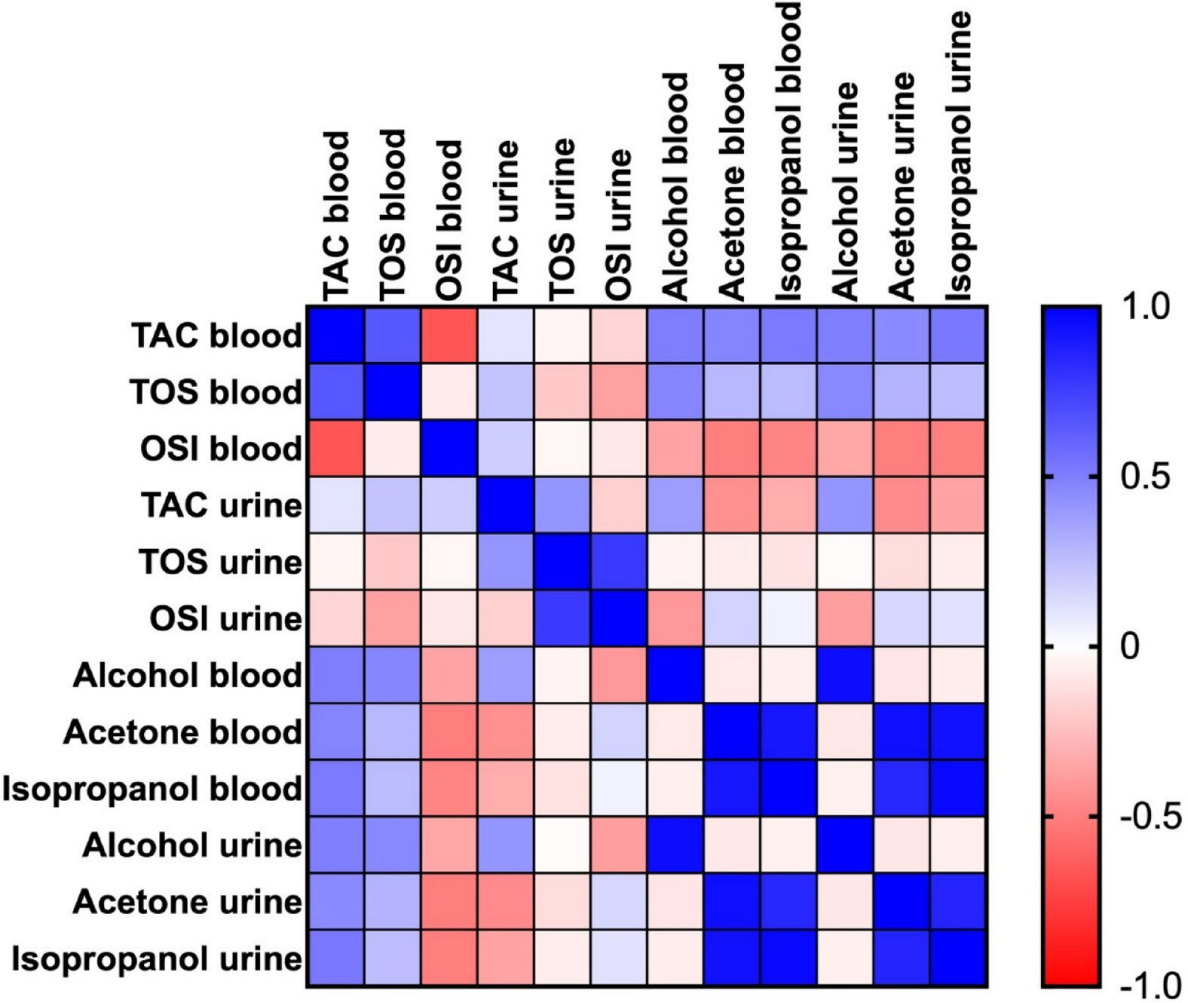
Heat map of correlations between redox status of blood and urine as well as blood and urinary alcohol, acetone, and also isopropanol levels. OSI: oxidative stress index; TAC: total antioxidant capacity; TOS: total oxidant status.

The results of the correlation analysis of the redox status with alcohol, acetone, and isopropanol concentrations in the blood and urine are shown in Fig. 3.

The level of the TAC in the blood was positively correlated with the blood TOS concentration (r = 0.654, *p* < 0.0001). However, it was negatively related to the blood OSI value (r = − 0.67, *p* < 0.0001).

The blood TOS concentration showed a negative correlation with the urinary OSI value (r = − 0.369, *p* = 0.005).

The blood TAC concentration was positively correlated with the blood alcohol (r = 0.504, *p* < 0.0001), acetone (r = 0.476, *p* < 0.0001), and isopropanol (r = 0.514, *p* < 0.0001), as well as with the urinary alcohol (r = 0.496, *p* < 0.0001), acetone (r = 0.454, *p* = 0.000189), and isopropanol (r = 0.523, *p* < 0.0001) levels. The blood TOS concentration was positively correlated with the blood alcohol (r = 0.472, *p* = 0.000108), acetone (r = 0.278, *p* = 0.029), and isopropanol (r = 0.263, *p* = 0.039), as well as with the urinary alcohol (r = 0.46, *p* = 0.000169), acetone (r = 0.297, *p* = 0.019), and isopropanol (r = 0.253, *p* = 0.048) concentrations. The blood OSI value was negatively correlated with the blood alcohol (r = − 0.357, *p* = 0.006), acetone (r = − 0.508, *p* < 0.0001), and isopropanol (r = − 0.482, *p* = 0.000127), as well as with the urinary alcohol (r = − 0.344, *p* = 0.008), acetone (r = − 0.508, *p* < 0.0001), and isopropanol (r = − 0.503, *p* < 0.0001) concentrations.

The urinary TAC concentration was positively correlated with the urinary TOS concentration (r = 0.415, *p* = 0.00145). The urinary TOS concentration showed a positive correlation with the urinary OSI value (r = 0.77, *p* < 0.0001).

The urinary TAC concentration had a positive correlation with the alcohol concentration in the blood (r = 0.381, *p* = 0.000289) and in the urine (r = 0.413, *p* = 0.00116). Nevertheless, the biomarker was negatively correlated with the blood and urinary acetone levels (r = − 0.437, *p* = 0.00053, and r = − 0.453, *p* = 0.00031, respectively), as well as with the blood and urinary isopropanol levels (r = − 0. 319, *p* = 0.01379, and r = − 0.36, *p* = 0.00505, respectively). The urinary OSI value was negatively correlated with the alcohol concentration in the blood (r = − 0.405, *p* = 0.002) and in the urine (r = − 0.384, *p* = 0.004).

The blood alcohol concentration positively correlated with its level in the urine (r = 0.951, *p* < 0.0001). The content of acetone in the blood showed a positive correlation with the blood isopropanol (r = 0.911, *p* < 0.0001), urinary acetone (r = 0.94, *p* < 0.0001), as well as with the urinary isopropanol (r = 0.931, *p* < 0.0001) concentrations. The blood isopropanol level was positively correlated with the urinary content of acetone (r = 0.845, *p* < 0.0001) and isopropanol (r = 0.961, *p* < 0.0001), too.

The isopropanol concentration in the urine positively correlated with the urinary level of that parameter (r = 0.855, *p* < 0.0001).

### ROC analysis

**Fig. 4:**
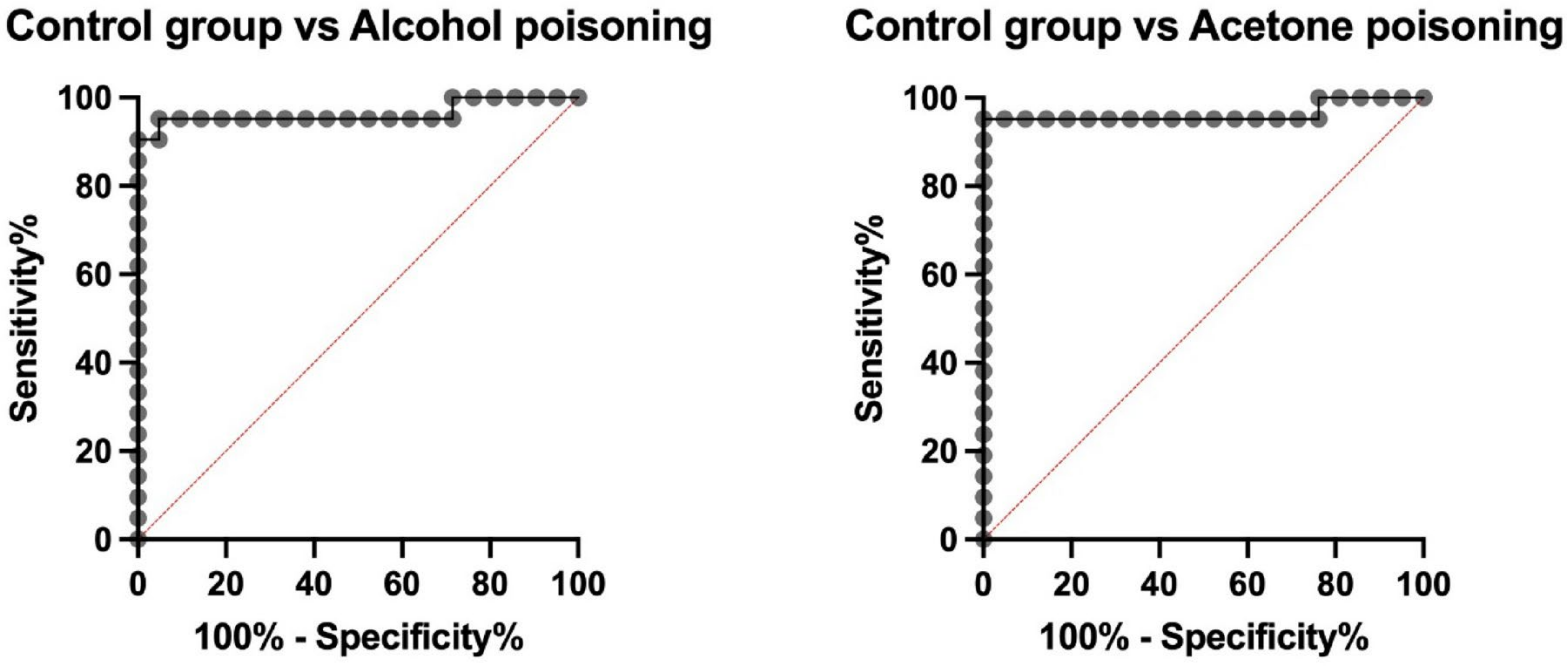
The area under the curve (AUC) for the blood total antioxidant capacity (TAC) concentration between the control and both the alcohol- and acetone-poisoned individuals.

The ROC analysis has revealed that the blood TAC concentration above 55.12 nmol/mg of protein had the AUC = 0.96 (*p* < 0.0001), effectively differentiating between the control and alcohol-intoxicated cadavers. The TAC blood concentration above 69.45 nmol/mg of protein also had the AUC = 0.96 (*p* < 0.0001), allowing for the distinction between the control group from the acetone-poisoned individuals (Table 2; Fig. 4).

**Table 2:** Results of the receiver operating characteristic (ROC) analysis of the total antioxidant capacity (TAC) in the blood between the control and both the alcohol- as well as acetone-poisoned cadavers.

	Blood TAC	
	Control vs. Alcohol poisoning	Control vs. Acetone poisoning
AUC	0.96	0.96
95% CI	0.9–1	0.89–1
Cut off	> 55.12	> 69.45
Sensitivity	95.24%	95.24%
95% CI	77.33–99.76%	77.33–99.76%
Specificity	95.24%	100%
95% CI	77.33–99.76%	84.54–100%

*AUC* area under the curve, *CI* confidence interval, *TAC* total antioxidant capacity.

## Discussion

Ethanol and acetone share several common mechanisms of toxicity. Both substances depress the CNS, leading to an impaired motor coordination, altered consciousness, and a potential respiratory failure. Ethanol and acetone also significantly disrupt the redox balance^7,28^. Ethanol, through metabolism to acetaldehyde, leads to an increase in the NADH/NAD + ratio, which impairs the mitochondrial function and energy production^29^. Acetone is a ketone body metabolized to acetoacetate and β-hydroxybutyrate, which leads to ROS overproduction responsible for lipid peroxidation, protein denaturation, and nucleic acid mutations^14,30^. However, the exact effects of acute alcohol and acetone intoxication on the systemic redox homeostasis have not yet been studied. In particular, no studies have compared the oxidoreductive balance in the acetone and alcohol-poisoned individuals. Little is also known about the use of redox biomarkers for assessing the alcohol exposure. There are no studies on acetone intoxication. Peter et al. revealed significantly lower blood and salivary TAC in male chronic alcoholics compared to age-matched non-alcoholic male volunteers^31^. Jun et al. reported that regular alcohol drinkers (who drank alcohol more than once per month) had meaningfully lower energy-adjusted dietary TAC as compared to non-drinkers^32^. In our previous study, we showed that TAC levels were statistically higher in the urine, but not in the blood, vitreous body, or cerebrospinal fluid of people who had died from acute ethanol poisoning^22^. However, in chronic alcohol users, we have shown that blood TAC can be a potential biomarker of alcohol consumption. The increase in TAC relative to TOS may be an adaptive response to ROS overproduction associated with alcohol exposure^23^. On the other hand, Henríquez-Sánchez et al. found that subjects in the highest quintile of the dietary total antioxidant capacity consumed more alcohol^33^. In contrast, consumption of alcohol (at least twelve drinks/year) did not significantly affect energy-adjusted dietary TAC levels^34^.

This study is the first to evaluate the blood and urine total redox status for assessing ethanol and acetone toxicity. The subjects who had died from alcohol and acetone poisoning and those who had died of sudden death (control) were included in the study. We have shown the blood TAC and TOS levels to be significantly higher while the blood OSI was significantly lower in the alcohol and acetone-intoxicated subjects as compared to the control group. The TAC assesses the overall, combined ability of various antioxidants to neutralize ROS and prevent cellular oxidative damage. In our assay, the blood TAC consists of protein thiols (52.9%), uric acid (33.1%), vitamin C (4.7%), bilirubin (2.4%), vitamin E (1.7%), as well as other antioxidants (5.2%)^15,26^. The TOS assesses the sum of ROS and other oxidants, while the OSI (TOS/TAC ratio) includes both the oxidative and antioxidant capacity, providing an accurate assessment of the oxidative stress level^19,25,27^. Therefore, the results of our study indicate a strengthening of the circulating antioxidant barrier under the influence of alcohol and acetone. It may represent an adaptive response to an increased ROS production in the people who died from alcohol and acetone poisoning. It is well known that an increase in the cellular antioxidant defense is a primary protective mechanism against oxidative stress in living systems^35–38^. Significant increases in the blood TAC in the study groups may be clinically relevant. The ROC analysis shows that the TAC distinguishes the ethanol- and acetone-poisoned individuals (AUC = 0.96 for both) from the control group with high sensitivity (both 95.24%) as well as specificity (95.24% and 100%, respectively). The blood TAC has proven to be also positively correlated with alcohol (r = 0.504, *p* < 0.0001), acetone (r = 0.476, *p* < 0.0001), and isopropanol blood levels (r = 0.514, *p* < 0.0001). The high AUC, sensitivity, and specificity values of the blood TAC as well as the strong correlation with the alcohol, acetone, and isopropanol levels reinforce its potential use in the clinical setting. Future research should focus on validating the blood TAC in larger, more diverse populations and exploring the integration of redox biomarkers into the clinical practice for the management of acute poisonings. However, it is important to remember that the TAC and TOS cannot reveal accurate information about respective antioxidants or oxidants in terms of the oxidative stress pathology. Furthermore, those biomarkers may be influenced by variables, including food, lifestyle, and general health, which may skew the results^27,39^.

In healthy individuals, the blood TAC and TOS highly correlate with their content in other circulating fluids^25,40–42^. In our study, the TAC, TOS, and OSI in the blood are poorly correlated with their urine concentrations, which may indicate differences in the redox profile between the studied bioliquids. Indeed, the urinary TAC was significantly higher in those who died from alcohol intoxication as compared to the acetone-poisoned and healthy individuals. The urinary TOS did not significantly differ among the groups, while the OSI was statistically lower in the alcohol-intoxicated patients as compared to the acetone-poisoned and healthy subjects. Those results indicate a significant enhancement of the antioxidant defense in the urine of the alcohol-poisoned patients. In terms of organ toxicity, ethanol’s most profound effects are observed in the liver (e.g., fatty liver disease, hepatitis, cirrhosis, and liver failure) and kidneys (e.g., acute and chronic kidney disease, hypertension, metabolic acidosis, dehydration, and electrolyte imbalance)^28,43^. Acetone shows a broader spectrum of organ involvement. Acetone primarily causes toxicity in the CNS and cardiovascular system. Acute acetone poisoning leads to metabolic acidosis, which severely disrupts the cardiovascular function, potentially resulting in arrhythmias or even heart failure. In rare cases, a prolonged exposure to acetone has also been associated with renal injury, although it is less common as compared to the ethanol-induced injury^7,10,44^. Therefore, the high urinary TAC in the alcohol-poisoned individuals may be due to alcohol nephrotoxicity. As with other organs, oxidative stress is one of the main mediators of ethanol nephrotoxicity^45,46^. The proximal tubule is particularly susceptible to the damage due to its high metabolic activity and a significant role in the reabsorption of solutes, electrolytes, and organic substances^46,47^. The metabolism of ethanol in the kidney (especially through ADH and cytochrome P450 2E1) generates superoxide radicals, hydrogen peroxide, and other ROS^46–48^. The high ROS levels lead to the mitochondrial dysfunction, impairing the energy production in the proximal tubule cells. Although the distal tubule and collecting duct are less metabolically active than the proximal tubule, ethanol also has a negative effect on them^45,46,49^. Ethanol directly inhibits the vasopressin secretion, reducing the ability of the collecting ducts to reabsorb water. It leads to diuresis and dehydration. Chronic exposure to ethanol may alter the electrolyte balance, leading to hypokalemia or hyponatremia^45,46,49^. Distal segments may also undergo oxidative damage, especially as a result of systemic effects of ethanol, such as dehydration and acid–base imbalances. In addition, the ethanol-induced oxidative stress may lead to glomerular damage by promoting mesangial cell contraction and increasing glomerular basement membrane permeability^45–49^.

Urine indicates the functional status of the kidneys and urinary tract^50,51^. The main component of the urinary TAC is uric acid^41^ which removes singlet oxygen, hydroxyl radicals, and peroxyl radicals. However, at high concentrations, uric acid has strong pro-oxidant effects^52–54^. Despite the increase in the urinary TAC in the alcohol poisoning group, the OSI was significantly lower as compared to the acetone poisoning and healthy subjects. It indicates a shift in the redox homeostasis in favor of antioxidant processes. The urinary TAC showed a positive correlation with the urine alcohol concentration (r = 0.413, *p* = 0.00116). However, the biomarker was negatively correlated with the acetone (r = − 0.453, *p* = 0.00031) and isopropanol urine levels (r = − 0.36, *p* = 0.00505, respectively). The urinary OSI value was also negatively correlated with the urine alcohol concentration (r = − 0.384, *p* = 0.004). It should be noted that urinary alcohol concentrations were higher in the people who had died from alcohol poisoning than in the blood, suggesting that ethanol accumulates in the kidneys during intoxication. It is confirmed by the results of other studies. In the case of the fatal alcohol poisoning, kidney damage is more severe than brain or eye damage^55^. Ethanol in the urine may be detected even a few hours longer than in the blood due to ethanol retention in the bladder, which also seems to be significant^56,57^. Further studies are required to assess the relationship between the urinary TAC and renal function parameters. Compared to blood collection, urine collection is less invasive and needs less work, tools, and skilled workers. Large volumes of urine may be collected several times a day. Urine contains fewer ROS promoters as well as lower levels of organic and inorganic metals. Thus, urine is less prone to oxidation and a more stable biological material^16,58,59^.

## Limitations and future prospects

It is important to acknowledge the limitations, strengths, and future directions of this study. According to Polish law, a forensic autopsy can only be performed 12 h after death has been confirmed. Although the study material was collected uniformly from all patients, the processes of body decomposition, including oxidation, can affect the redox homeostasis of circulating fluids^60^. Within a few hours of death, blood clotting and clot lysis occur, and autolysis begins, primarily due to endogenous and exogenous bacteria as well as various enzymes^61,62^. Therefore, the results presented here should be interpreted with consideration of post-mortem processes. However, there is still limited knowledge about the impact of body decomposition on antioxidant systems and oxidative stress in cadavers, highlighting the need for further research. Future studies should also focus on standardizing measurement methods, evaluating specific antioxidants and oxidants, and verifying TAC, TOS and OSI within larger and more diverse populations. While the number of patients in this study is typical for forensic medicine, it is crucial to assess how comorbidities, diet, and medications may influence the redox homeostasis of individuals fatally poisoned with alcohol and acetone. It is well known that infections, chronic diseases, and drugs can interfere with measurements performed using Erel’s method^15,25,26^. Additionally, redox homeostasis is influenced by age and gender, which is why the control group was matched to the study groups.

## Conclusions

In conclusion, we have shown that fatal alcohol and acetone intoxications disrupt the circulating redox status in favor of antioxidant reactions. We highlight the disruption of blood and urine redox homeostasis caused by differences in organ sensitivity to alcohol and acetone toxicity. The TAC, TOS, and OSI concentrations in the blood and urine correlate highly with the alcohol, acetone, and isopropanol levels. With high sensitivity and specificity, the blood TAC differentiates alcohol and the acetone-poisoned individuals from healthy controls.

## Electronic supplementary material

Below is the link to the electronic supplementary material.

Supplementary Material 1

## Data Availability

The data that support the findings of this study are included in the manuscript and in the supplementary material.

## Abbreviations

ABTS: 2,2′-Azino-bis(3-ethylbenz-thiazoline-6-sulfonic acid)
ABTS^·+^: Cationic radical 2,2′-azino-bis(3-ethylbenz-thiazoline-6-sulfonic acid)
ADH: Alcohol dehydrogenase
ALDH: Aldehyde dehydrogenase
AUC: Area under the curve
BCA: Bicicinchoninic acid
CNS: Central nervous system
FID: Flame ionization detection
GABA: Gamma-aminobutyric acid
HS-GC: Headspace gas chromatography
H_2_O_2_: Hydrogen peroxide
H_2_SO_4_: Sulfuric acid
NaCl: Sodium chloride
NPDS: National poison data system
OSI: Oxidative stress index
ROC: Receiver operating characteristic
ROS: Reactive oxygen species
TAC: Total antioxidant capacity
TOS: Total oxidative status
Trolox: 6-Hydroxy-2,5,7,8-tetramethylchroman-2-carboxylic acid
