# Supplementary material for: Assessment of blood and urine total antioxidant and oxidative status in alcohol and acetone fatal poisonings
Source: Sci Rep. 2025 Jul 7;15:24163. doi: 10.1038/s41598-025-09494-x (PMC12234996; doi:10.1038/s41598-025-09494-x)
Supplement: Supplementary file 1 — Supplementary Material 1 [file 41598_2025_9494_MOESM1_ESM.pdf]

Sex	Age	Cause of death	Alcohol blood	Acetone blood	Isopropanol blood	Alcohol urine	Acetone urine	Isopropanol urine
male	35	chest injuries	0	0	0	0	0	0
male	37	brain injuries	0	0	0	0	0	0
female	55	brain injuries	0	0	0	0	0	0
male	53	brain injuries	0	0	0	0	0	0
male	57	brain injuries	0	0	0	0	0	0
female	27	chest injuries	0	0	0	0	0	0
male	35	brain injuries	0	0	0	0	0	0
female	51	chest injuries	0	0	0	0	0	0
male	34	brain injuries	0	0	0	0	0	0
male	67	brain injuries	0	0	0	0	0	0
male	56	brain injuries	0	0	0	0	0	0
male	32	chest injuries	0	0	0	0	0	0
male	32	brain injuries	0	0	0	0	0	0
male	48	chest injuries	0	0	0	0	0	0
male	54	brain injuries	0	0	0	0	0	0
male	63	brain injuries	0	0	0	0	0	0
female	59	brain injuries	0	0	0	0	0	0
female	51	brain injuries	0	0	0	0	0	0
male	35	chest injuries	0	0	0	0	0	0
male	57	brain injuries	0	0	0	0	0	0
male	37	brain injuries	0	0	0	0	0	0
male	37	alcohol intoxication	4	0	0	4.7	0	0
female	27	alcohol intoxication	4	0	0	5	0	0
female	51	alcohol intoxication	4.1	0	0	4.6	0	0

male	56	alcohol intoxication	4	0	0	4	0	0
male	32	alcohol intoxication	4.2	0	0	4.4	0	0
male	63	alcohol intoxication	4.1	0	0	4.2	0	0
male	35	alcohol intoxication	4.3	0	0	4.4	0	0
male	57	alcohol intoxication	4.7	0	0	4.9	0	0
female	55	alcohol intoxication	4	0	0	4.1	0	0
female	59	alcohol intoxication	4.6	0	0	6.1	0	0
male	57	alcohol intoxication	5	0	0	6.2	0	0
male	54	alcohol intoxication	4	0	0	4.1	0	0
male	35	alcohol intoxication	4.1	0	0	4.2	0	0
female	51	alcohol intoxication	4	0	0	5.1	0	0
male	35	alcohol intoxication	4.4	0	0	4.5	0	0
male	53	alcohol intoxication	4.1	0	0	4.3	0	0
male	37	alcohol intoxication	4	0	0	4.3	0	0
male	48	alcohol intoxication	4.5	0	0	5.1	0	0
male	32	alcohol intoxication	4	0	0	4.1	0	0
male	34	alcohol intoxication	4.2	0	0	4.2	0	0
male	67	alcohol intoxication	4.3	0	0	4.7	0	0
female	55	acetone intoxication	1.4	0.56	0.13	2.4	0.74	0.14
male	32	acetone intoxication	2.3	0.6	0.6	2.6	0.75	0.75
male	54	acetone intoxication	1.3	0.58	0.5	2.8	0.79	0.74
male	35	acetone intoxication	1.2	0.57	0.12	2	0.73	0.18
female	51	acetone intoxication	0.4	0.55	0	1.1	0.81	0
male	56	acetone intoxication	0.3	0.59	0	0.4	0.83	0
male	63	acetone intoxication	0.3	0.56	0	1.4	0.64	0.06
female	59	acetone intoxication	2.3	0.6	0.15	0.8	0.6	0.23

male	48	acetone intoxication	0	0.6	0.36	0.01	0.6	0.1
male	37	acetone intoxication	0.2	0.6	0.07	1.7	0.6	0.08
male	35	acetone intoxication	1.9	0.6	0.6	2.9	1.57	0.11
male	34	acetone intoxication	2.5	2.35	0.33	1.7	0.56	0.3
male	37	acetone intoxication	0.1	0.57	0.17	0.4	0.73	0.19
male	57	acetone intoxication	0	0.63	0.13	0	0.75	0.23
male	32	acetone intoxication	1.6	0.71	0.4	0	0.86	0.13
male	57	acetone intoxication	0	0.64	0.13	0	0.97	0.17
male	67	acetone intoxication	0	0.58	0.08	0	0.72	0.18
female	51	acetone intoxication	3	0.78	0.58	3.1	0.55	0.9
male	35	acetone intoxication	0.1	0.61	0.05	0.6	0.62	0.16
female	27	acetone intoxication	0.5	0.66	0.12	1	0.7	0.26
male	53	acetone intoxication	0	0.56	0.04	0.7	0.6	0.08
